# Regulation of electron transfer processes affects phototrophic mat structure and activity

**DOI:** 10.3389/fmicb.2015.00909

**Published:** 2015-09-03

**Authors:** Phuc T. Ha, Ryan S. Renslow, Erhan Atci, Patrick N. Reardon, Stephen R. Lindemann, James K. Fredrickson, Douglas R. Call, Haluk Beyenal

**Affiliations:** ^1^The Gene and Linda Voiland School of Chemical Engineering and Bioengineering, Washington State University, Pullman, WAUSA; ^2^Environmental Molecular Sciences Laboratory, Pacific Northwest National Laboratory, Richland, WAUSA; ^3^Biological Sciences Division, Pacific Northwest National Laboratory, Richland, WAUSA; ^4^Paul G. Allen School for Global Animal Health, Washington State University, Pullman, WAUSA

**Keywords:** electron transfer, Hot Lake, microbial mats, current, microelectrodes, mat structure, metabolite analysis, microbial community

## Abstract

Phototrophic microbial mats are among the most diverse ecosystems in nature. These systems undergo daily cycles in redox potential caused by variations in light energy input and metabolic interactions among the microbial species. In this work, solid electrodes with controlled potentials were placed under mats to study the electron transfer processes between the electrode and the microbial mat. The phototrophic microbial mat was harvested from Hot Lake, a hypersaline, epsomitic lake located near Oroville (Washington, USA). We operated two reactors: graphite electrodes were polarized at potentials of -700 mV_Ag/AgCl_ [cathodic (CAT) mat system] and +300 mV_Ag/AgCl_ [anodic (AN) mat system] and the electron transfer rates between the electrode and mat were monitored. We observed a diel cycle of electron transfer rates for both AN and CAT mat systems. Interestingly, the CAT mats generated the highest reducing current at the same time points that the AN mats showed the highest oxidizing current. To characterize the physicochemical factors influencing electron transfer processes, we measured depth profiles of dissolved oxygen (DO) and sulfide in the mats using microelectrodes. We further demonstrated that the mat-to-electrode and electrode-to-mat electron transfer rates were light- and temperature-dependent. Using nuclear magnetic resonance (NMR) imaging, we determined that the electrode potential regulated the diffusivity and porosity of the microbial mats. Both porosity and diffusivity were higher in the CAT mats than in the AN mats. We also used NMR spectroscopy for high-resolution quantitative metabolite analysis and found that the CAT mats had significantly higher concentrations of osmoprotectants such as betaine and trehalose. Subsequently, we performed amplicon sequencing across the V4 region of the 16S rRNA gene of incubated mats to understand the impact of electrode potential on microbial community structure. These data suggested that variation in the electrochemical conditions under which mats were generated significantly impacted the relative abundances of mat members and mat metabolism.

## Introduction

Phototrophic microbial mats are remarkable self-sustaining natural ecosystems, being composed of highly interactive species that completely cycle energy and elements within them (Guerrero et al., 2002; Des Marais, 2003; Bender and Phillips, 2004). These systems undergo daily cycling of redox potential caused by variations in light energy input and metabolic interactions among the microbial species (Frund and Cohen, 1992; Burow et al., 2012). Light also drives mat structure and activity by providing a diverse energy spectrum to sustain photosynthesis in photosynthetic microorganisms, which fix carbon and produce organic materials needed by other microorganisms in the community. Numerous studies have characterized the variations in physicochemical parameters, the elemental cycles, and the diversity and interactions of the microbial community members within the mat (Des Marais, 1995; Decker et al., 2005; van der Meer et al., 2005; Burow et al., 2012; Lindemann et al., 2013; Pages et al., 2014). Mat systems provide an excellent ecological model that can be used to investigate how microbial populations associate and interact as well as how electrons are used for energy transfer (Guerrero et al., 2002; Babauta et al., 2014). In this ecological model, electrons have to be transferred to convert and carry captured light energy to the deeper layers of the mat. The mat structure, inclusive of its physical structure and pore distribution, may play a critical role in these mass and energy transport processes (Moran et al., 2014). Therefore, it is critical to understand the effects of electron transfer processes on mat structure and activity. Energy transfer in a microbial mat starts with light energy being absorbed in the photic zone of the mat, which allows fixation of carbon dioxide by photoautotrophs. This energy transfer is mediated by diverse processes, such as the synthesis of organic compounds (e.g., carbohydrates) and their diffusion to deeper, and frequently hypoxic, strata of the mat where they may be consumed by fermenters and/or sulfate reducers (Paerl et al., 2000). Many of these reactions involve extracellular electron transfer processes, which can occur either directly or through electron carriers (mediators). We hypothesized that regulating the electron transfer processes in a mat system would, in turn, change its structure and activity. “Structure” refers to the physical structure or architecture of the mat, and “activity” refers to the metabolites and energy exchanged by the mat. According to our previous work and other work in the literature, electron transfer processes can be controlled by controlling the potential of an inert solid electrode and using it as an electron donor or acceptor in a biological system (Renslow et al., 2013).

Use of polarized electrodes to study electron transfer by microorganisms has become an important tool for filling the knowledge gap about microbial electrochemical activities (Rabaey et al., 2007). This technique offers a unique strategy for controlling the activity of a microbial population by accepting or donating electrons from/to the local environment. The measured electron transfer rate can be used to interpret the response of microbial activities to the perturbation of electron transfer processes. Some microbial species respond by altering their metabolism, activating a defense mechanism, or reducing their growth rates. Other microbial species benefit because of their ability to transfer or accept electrons from the electrodes (Babauta et al., 2014). Thus, by increasing the metabolic activities of certain species present near the electrode surface, the electrode potential will not only select the species enriched on it but also regulate the structure, activity, and composition of the local microbial community. To the best of our knowledge, the responses of mat structure, activity, and electron transfer rates when solid electrodes are employed to accept or donate electrons have not yet been quantified.

The goal of this work is to characterize the response of a microbial mat to a solid electron acceptor and a donor. The phototrophic microbial mats used in this study were derived from Hot Lake, a hypersaline, epsomitic lake located near Oroville (Washington, USA). The seasonal characteristics of mat and lake conditions were described detail by Lindemann et al. (2013). The electrodes were placed at the bottom of the mat to create the mat electrochemical systems. The electrodes that provided electrons to the mat were polarized at -700 mV_Ag/AgCl_ (hereafter called the “CAT mat”). The ones that worked as an electron sink were polarized at +300 mV_Ag/AgCl_ (hereafter called the “AN mat”). The electron transfer rates from mat-to-electrode and from electrode-to-mat were continuously monitored. We quantified the diel cycling of AN and CAT mats. To understand the factors affecting the diel cycle, (1) we examined the influences of temperature and light condition, separately, to electron transfer rate; (2) we used microelectrodes to measure the depth profiles of hydrogen sulfide and oxygen concentrations; and (3) we introduced acetate and sulfide into AN and CAT mats and quantified their influence on electron transfer rates, separately. To understand how AN or CAT processes regulate mat structure and activity, we used nuclear magnetic resonance (NMR) imaging for morphology and NMR metabolite analysis to quantify the activity of the mats. Finally, we employed amplicon sequencing across the V4 region of the 16S gene to determine the effect of electrochemical regime upon mat community structure.

## Materials and Methods

### Field Site and Sample Collection

Hot Lake is located near Oroville, North Washington, CO, USA. Photosynthetic microbial mats and Hot Lake water were sampled on June 6th and September 26th, 2014. Each mat sample was between 1.5 and 2 cm in depth and about 200 cm^2^ in size. The samples were kept in petri dishes and placed in a cold ice box when carried to our laboratory at Washington State University, Pullman, WA, USA. Upon arrival, the mat was paced in glass jars and covered with Hot Lake water from the field site. Mats were kept under natural solar conditions and at room temperature for 2 days before being transferred to the mat electrochemical systems.

### Construction of Mat Electrochemical Systems and Current Monitoring

**FIGURE 1:**
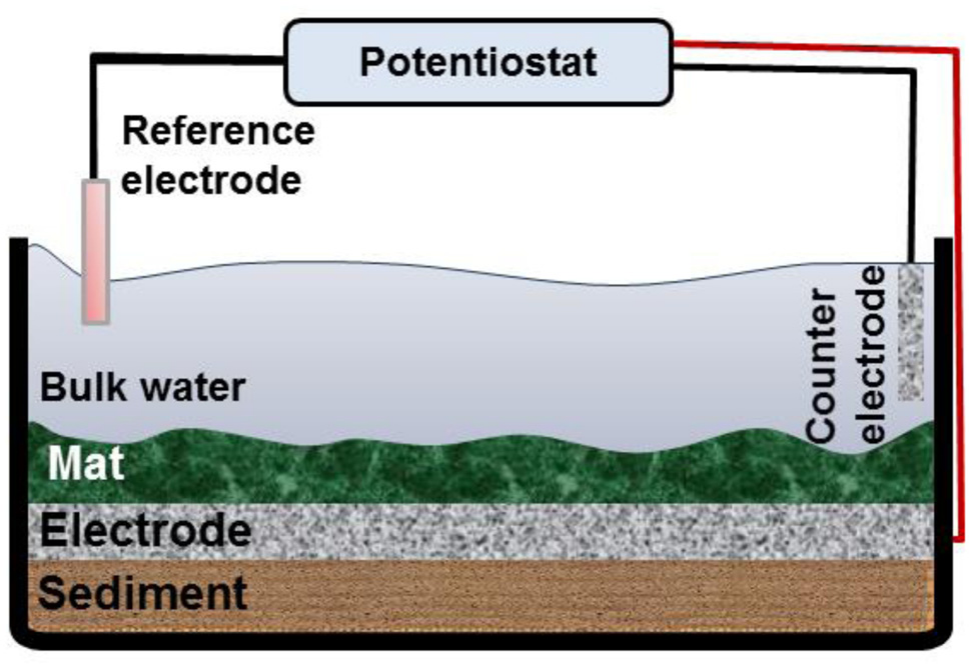
**Schematic diagram of the mat electrochemical systems.** The working electrode was placed right below the mat, and the counter electrode was located in the bulk solution. A potentiostat was used to control the working electrode potential vs. the Ag/AgCl reference electrode.

We used an acrylic aquarium with a working volume of 1.5 L to construct the mat electrochemical systems. The reactors mimicked the lake condition without flow (**Figure 1**). For each reactor, approximately 3 cm of sediment was added to the bottom of the reactor. Then the hollow polycarbonate plate on which the graphite cloth electrode (2.5c m × 2.5 cm) was embedded was placed on top of the sediment. The mat was carefully placed on top of the electrode. The electrode placed under the mat was used as the working electrode. The counter electrode was constructed by attaching another piece of graphite cloth (2.5 cm × 2.5 cm) to a hollow plastic dish and placing it in the water phase of the reactor. Ti wire was woven into each electrode and secured with nylon bolts. The part of the Ti wire that was exposed to Hot Lake water was covered by marine sealant and inserted into a sealed silicon tube. During operation of the reactor, deionized (DI) water was added periodically to maintain the water level and salinity in the reactor. All the reactors were operated under natural solar conditions: they were incubated near a laboratory window. For some experiments, the reactors were moved to an incubator or to a water bath with controlled temperature, as described in the relevant sections.

A 6-channel custom-built potentiostat was used to control the desired potential of the working electrodes under the mat (Renslow et al., 2011). The custom-made Ag/AgCl reference electrodes were used (~+210 mV vs. SHE). We operated reactors in which the graphite electrodes were polarized at potentials of -700 mV_Ag/AgCl_ (CAT mat) and +300 mV_Ag/AgCl_ (AN mat). These potentials are in the range that was previously used to enrich AN and CAT communities in bioelectrochemical systems (Wagner et al., 2010; Tremblay and Zhang, 2015). Moreover, due to the presence of sulfide in the mat we polarized cathode at lower potential than the redox potential of sulfide (E^o^_H2S/So_ = -490 mV_Ag/AgCl_) to prevent the deposition of elemental sulfur on the electrode. We also operated a mat system under open circuit condition as the control mat [open circuit potential (OCP) mat]. Two technical replicates with two biological replicates were performed for all experiments.

### Effects of Light Intensity, Temperature and Electron Donor/Acceptor Addition on the Mat

To examine the effects of light/dark conditions with AN and CAT currents, we operated our mat systems in an incubator. An incandescent light bulb was used as the light (light intensity of 6.4 ± 0.2 μmol/m^2^/s). The temperature was maintained by the incubator at the same value as under the light (22.5 ± 1°C). To examine the effect of temperature on current, the mat electrochemical systems were placed in a temperature-controlled water bath. The water bath was covered to prevent light penetration when needed. The effects of acetate and sulfide on the electron transfer process were examined by carefully injecting 2 mL of 1 M acetate solution or 0.3 mL of 0.1 M Na_2_S.9H_2_O solution into the bottom of the mat. The mat systems were operated under constant light (6.4 ± 0.2 μmol/m^2^/s) and stable temperature (22.5 ± 1°C) to minimize the acetate and sulfide production by the mat itself.

### Dissolved Oxygen and H_2_S Microelectrodes and Measurements

The DO microelectrodes were constructed according to the procedure described by Lewandowski and Beyenal (2014). The final tip diameter of the DO microelectrodes was ~15 μm. A Keithley 6517 A electrometer/high-resistance meter was used as both the voltage source and an ammeter. The DO microelectrodes were polarized to -800 mV_Ag/AgCl_ during the operation. Prior to use, the DO microelectrodes were calibrated using two-point calibration: in the air-saturated solution and in Na_2_SO_3_ solution (zero oxygen concentration). Microelectrode movements were administered using a Mercury Step motor controller PI M-230.10S Part No. M23010SX (Physik Instrumente, Auburn, MA, USA). Each microelectrode was positioned ~2000 μm above the mat surface and stepped down in 50-μm increments using a custom microprofiler. For the DO profiles, the measurements were taken in the air first (~1 mm) and in the bulk above the mat (~1 mm) until the microelectrode touched the mat surface; then ~3 mm of profile was measured in the mat. Before the DO profiles were measured, some of the bulk liquid above the mat was discharged.

The hydrogen sulfide microelectrodes were constructed and operated according to Jeroschewski et al. (1996). The hydrogen sulfide microelectrodes had tip diameters of ~20 μm. A Gamry Interface 1000TM potentiostat (Gamry^^®^^ Instruments, Warminster, PA, USA) was used to polarize the hydrogen peroxide microelectrodes at +100 mV against a Pt counter electrode. The microelectrode was calibrated in standard solutions of various hydrogen sulfide concentrations prepared by dissolving Na_2_S in anoxic phosphate buffer (16 mM, pH 7). The response of the microelectrodes was linear in the range of hydrogen sulfide concentrations from 0 to 570 μM. The hydrogen sulfide microelectrode was placed ~1 mm above the mat surface, and the microelectrode was stepped down in 1-mm increments through 13 mm of the mat thickness using the manual control. After the depth profiles were measured, the microelectrode was left at the 13-mm depth to monitor the hydrogen sulfide concentration over 24 h. Each microelectrode used in the measurements was also calibrated for verification after the measurements. A Zeiss Stemi 2000 stereomicroscope was used to determine the locations of the microelectrode tip and the mat surface during all microelectrode experiments.

### Morphology Analysis using NMR Micro-Imaging

Pulsed-field gradient NMR (PFG-NMR) was used to observe mat morphology and determine intra-mat porosity and diffusion coefficients. The techniques used were similar to those of Renslow et al. (2013). The details of the NMR imaging techniques and full method descriptions are provided in the Supplementary Information. Briefly, the NMR imaging experiments were conducted at 500.40 MHz for proton (1H) detection using a 89-mm-wide bore 11.7-T magnet with a Bruker Avance III digital NMR spectrometer and ParaVision 5.1 imaging software (Bruker Instruments, Billerica, MA, USA). Each mat sample was placed in a 15-mm NMR tube on a support bed of 2% agar gel. Experiments performed included 2D magnetic resonance imaging (mic_flash), diffusion tensor imaging for determining diffusion coefficients (DtiStandard; Renslow et al., 2013), and chemical shift selective imaging for generating porosity measurements.

### Metabolite Analysis using NMR Spectroscopy

Sections of microbial mat were dissected out such that the full thickness of the mat was retained. Mat samples were frozen at -80°C immediately following dissection and lyophilized before their dry weight was measured. Dried samples were coarsely ground in glass vials using a small metal spatula. Metabolite extraction was performed using a 1:1:1 volume of water:methanol:chloroform. Ice cold solvents were sequentially added to the samples in the order listed. Samples were allowed to extract and phase separate at -20°C for ~24 h. Samples were spun at 4000 × g for 10 min, and the hydrophilic supernatant was removed. Samples were spun again at 20800 × g for 10 min, and 1 mL of supernatant was retained. The retained samples were dried under vacuum in a speedvac overnight. Dried samples were dissolved in 50 mM sodium phosphate at pH 7, with 10% D_2_O, 448 uM DSS, and 10 mM imidazole.

Nuclear magnetic resonance spectroscopy was carried out using an Agilent VNMRS 600 MHz (proton frequency) spectrometer equipped with a cryogenically cooled HCN triple resonance probe. One-dimensional NOESY experiments were performed using the following parameters, 28896 complex points (acquisition time of 4 s), a recycle delay of 1 s, a sweep width of 12 ppm and 512 scans per experiment for a total acquisition time of ~48 min per sample. The temperature was held constant at 25°C. The NMR spectra were Fourier transformed, phased, baseline corrected and apodized (0.5-Hz line broadening) using nmrPipe. Spectra were referenced to DSS at 0 ppm. Data were imported into Chenomx NMR Suite (Chenomx, Inc., Edmonton, AB, Canada) for subsequent metabolic profiling. Profiling was carried out independently by two operators to reduce operator bias. The DSS internal standard was used to determine metabolite concentrations. Imidazole peaks were used to correct for pH differences. The raw data in varian format is submitted as Supplemental Information.

### Community Structure Analysis using 16S rRNA Sequencing

Mat community structure changes were examined using 16S rRNA amplicon sequencing. Two samples of mat (~4 cm^2^) were collected for each condition: (OCP, AN and CAT). These samples were further subdivided into nine subsamples. Genomic DNA (gDNA) was extracted as described in Lindemann et al. (2013) with the following modifications: prior to extraction, samples were washed three times with washing buffer containing 0.5 M EDTA and 0.55 M NaCl at pH 8.0 and suspended in lysis buffer (50 mM Tris/25 mM EDTA pH 8.0). Samples were then transferred to Lysing Matrix E tube (MP Biomedicals, Santa Ana, CA, USA) and beat for 2 min. After centrifugation (16,000 rpm for 90 s) the aqueous portions were incubated at 85° C for 5 min to inactivate native nucleases and slowly cooled to 37°C. Chemical lysis then was initiated by adding 70 μl of 10% SDS and proteinase K to the final concentration of 0.2 mg/ml prior to incubation at 56°C for 1 h. Subseqently, 100 μl of NaCl 5 M and 100 μl of CTAB/NaCl solution were added to samples and incubated at 65°C for 10 min. Post-lysis, DNA was extracted with phenol-chloroform-isoamyl alcohol (25:24:1) and then chloroform-isoamyl alcohol (24:1) before being treated with RNAase (10 μg) at 37°C for 30 min. DNA was precipitated with addition of 1:10 vol of sodium acetate 3 M (pH 5.5) and ice-cold 100% ethanol, washed in 70% ethanol, dried and resuspended in TE buffer (10 mM Tris-HCl at pH 8.0, 1 mM EDTA). Similarly, gDNA was extracted from fresh mats harvested at Hot Lake at the same time as those used in incubation studies but immediately frozen in 2.3 M sucrose and held at -80°C prior to extraction. gDNA was amplified and sequenced as described previously (Caporaso et al., 2012). Briefly, the V4 region of the 16S rRNA gene was amplified using primers 515F and 806R with 0–3 random bases and the Illumina sequencing primer binding site (Caporaso et al., 2011) and amplicons were sequenced on an Illumina MiSeq using MiSeq Reagent Kit MS-102-2001 {[v2 chemistry over 500 total cycles (2 × 250); Illumina, San Diego, CA, USA]}. Sequences were demultiplexed and processed using mothur v. 1.34.1 (Schloss et al., 2009) according to the MiSeq SOP (http://mothur.org/wiki/MiSeq_SOP, accessed 7/6/15) except for the following modifications: (1) sequences were aligned against a SILVA reference alignment to which full-length 16S clones from Hot Lake had been added (Lindemann et al., 2013), and (2) only sequences that could not be placed within any of the domains of life were removed using remove.lineage(taxon = unknown). Sequences were clustered using a 0.03 average neighbor cutoff using cluster.split at the Order level (taxlevel = 4) and subsampled to 6283 sequences per sample; all samples containing fewer sequences were discarded from the analysis. Bray–Curtis dissimilarity (Bray and Curtis, 1957) was calculated for all pairwise combinations of samples using dist.shared (calc = braycurtis) and a neighbor-joining dendrogram was constructed using the tree.shared() command.

## Results and Discussion

### Electron Transfer Rates in Anodic and Cathodic Mat Systems

**FIGURE 2:**
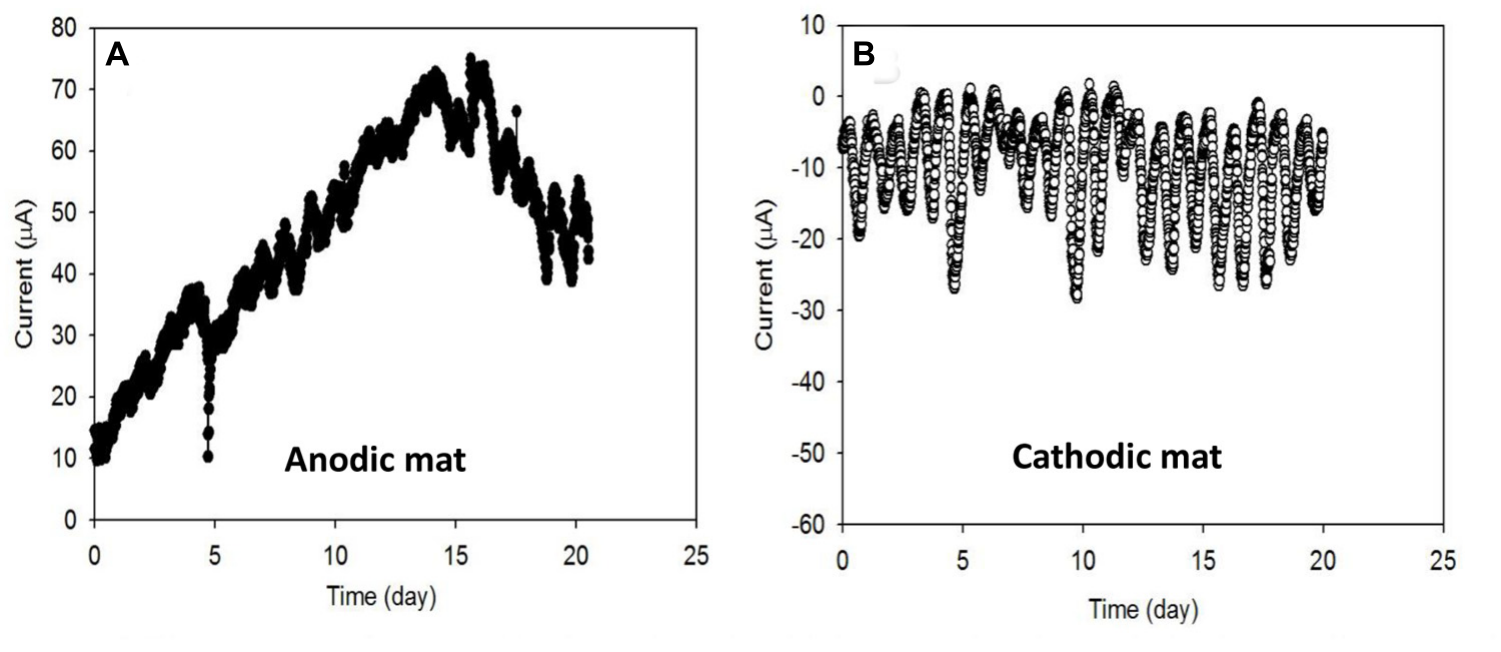
**Electron transfer rates **(A)** from the microbial mat to the electrode in the anodic (AN) mat and **(B)** from the electrode to the microbial mat in the cathodic (CAT) mat.** Both systems showed the cyclic fluctuation of current which is also referred to as the diel cycle.

Current generated from AN and CAT mats over the first 20 days under ambient light conditions is shown in **Figure 2**. Increasing AN current over time (**Figure 2A**) indicated that electrons were transferred from the mat to the solid electrode during the first 15 days. The current generation started immediately after the electrode was polarized. It developed in the range from 10 μA to about 70 μA with cyclic fluctuation. We hypothesized that these electrons may be transferred to the anode either (1) through direct sulfide oxidation (Rabaey et al., 2006; Babauta et al., 2014) and/or (2) through microbial exogenous electron transfer (Lovley, 2008; Logan, 2009). In the first case, the abiotic oxidation of sulfide generates electrons and elemental sulfur is deposited on the electrode (Rabaey et al., 2006). Sulfide is commonly produced in phototrophic mats by sulfate-reducing bacteria. Because the sulfate concentration in the Hot Lake environment is extremely high, ranging from ~200 mM to 1.8 M (Lindemann et al., 2013), the rate of sulfide production depends on the availability of substrates for sulfate reducers such as H_2_ and acetate. In the case of exogenous electron transfer, electrogenic bacteria act as a catalyst which oxidizes substrates and transfers electrons to the electrode. The rate of electron transfer is governed by the enrichment of the electrogens and the concentration of the available substrates in the mat. Thus, in both cases the current generated by an AN microbial mat is regulated by substrate availability at the bottom of the mat.

A recent study reported that green sulfur bacteria were a dominant population on a polarized AN microelectrode (Babauta et al., 2013). Sulfur cycling in combination with the activities of green sulfur bacteria was hypothesized to be the cause for the cycling of current. In another study, Badalamenti et al. (2013) used a large-size graphite electrode to enrich AN biofilm from a microbial mat inoculum (Badalamenti et al., 2013). The authors found that the phototrophically enriched anode biofilms were dominated by green sulfur bacteria. These bacteria were reported to have the ability to respire on electrodes. Thus, it is possible that the phototrophic community is also involved in the current production from our AN mat.

We also observed that the negative current generation from the CAT mat started immediately after the electrode was polarized (**Figure 2B**). Like the AN condition, mat under CAT conditions showed cyclic current fluctuations. Among the cycles, the lowest current ranged from approximately -0 μA to approximately -5 μA while the highest ranged from about -10 μA to about -30 μA. The generation of negative current indicates the consumption of electrons from the solid electrode by the mat. These electrons could be consumed by (1) an abiotic oxidant such as oxygen or (2) biocathodic microbes (Lovley and Nevin, 2011; Rosenbaum et al., 2011). In the first case, the current would be controlled by the DO concentration near the electrode surface. Oxygen is generated in the mat during the day by oxygenic phototrophs in the illuminated zone near the surface. Thus, the abiotic CAT current will be ultimately governed by photosynthetic activity and the diffusion of the oxygen through the mat. In the latter case, the current would be governed by the enrichment of microbes that are capable of using the electrode as an electron source for their metabolism.

In the literature, CAT current has been obtained from mixed CAT cultures enriched from wastewater (Marshall et al., 2012), activated sludge (Su et al., 2013), freshwater (Babauta et al., 2013), and pure bacterial cultures (Gregory et al., 2004; Nevin et al., 2011; Ueki et al., 2014). Our study demonstrates that CAT current can also be generated by the phototrophic mat community. Microbes that can potentially consume electrons from solid electrodes include methanogens (Villano et al., 2010), acetogens (Nevin et al., 2011), phototrophs (Bose et al., 2014) and oxygen reducers (Clauwaert et al., 2007; Debuy et al., 2015).

### Diel Cycle of Cathodic and Anodic Current

**FIGURE 3:**
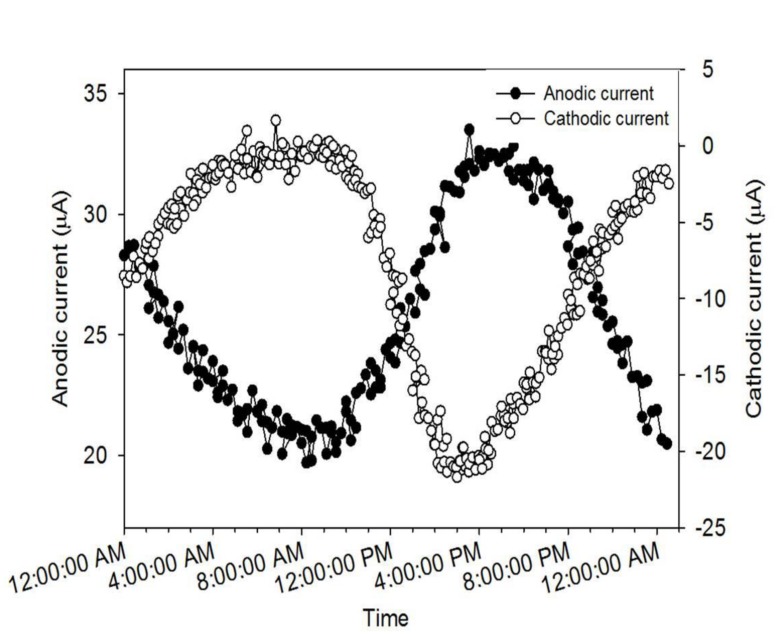
**Diel cycle of AN and CAT current generated from microbial mat systems**.

To understand the reasons for the cyclic fluctuation seen during the development of AN and CAT current, we superimposed individual cycles of each current as a function of time. **Figure 3** shows that both AN and CAT current followed an oscillating pattern in tandem with the diel cycle. It is known that phototrophic mats generally undergo a diel cycling of redox potential and speciation of redox-active elements caused by diel variations in light intensity and temperature and hence metabolic interactions among the microbial species (Finke and Jorgensen, 2008; Burow et al., 2012). Thus, the diel cycling of our current is likely to have been caused by these diel variations.

Interestingly, the lowest and highest points in the diel cycles of AN and CAT current occurred at the same time (**Figure 3**). The minimum values of AN and CAT current both occurred at about 8:00 a.m. (20 μA for AN current and -1 μA for CAT current). Similarly, the AN mat generated its highest current (33 μA) simultaneously with the CAT mat generating its highest current (-22 μA), at around 4:00 p.m. Thus, the CAT mat achieved its highest reducing potential at the same time that the AN mat achieved its highest oxidizing potential. The similarity in diel pattern suggested that the CAT and AN mat currents were governed by the same factors. However, the contrary redox characteristics of the two mat systems indicated that (1) there was an element or a microbial group in the mat that could function as both electron donor and electron acceptor to/from a solid electrode or (2) the characteristic of the mat was changed by the polarized potential of the solid electrode.

### Effect of Temperature on Electron Transfer Process

Under natural conditions, temperature and light intensity co-vary. Therefore, to isolate the effect of temperature change on the current, we operated the system in the dark. The AN and CAT currents were compared with the maximum current obtained at 4:00 p.m. from the closest diel cycle. At that time, the temperature was about 21.3 ± 0.2°C.

**FIGURE 4:**
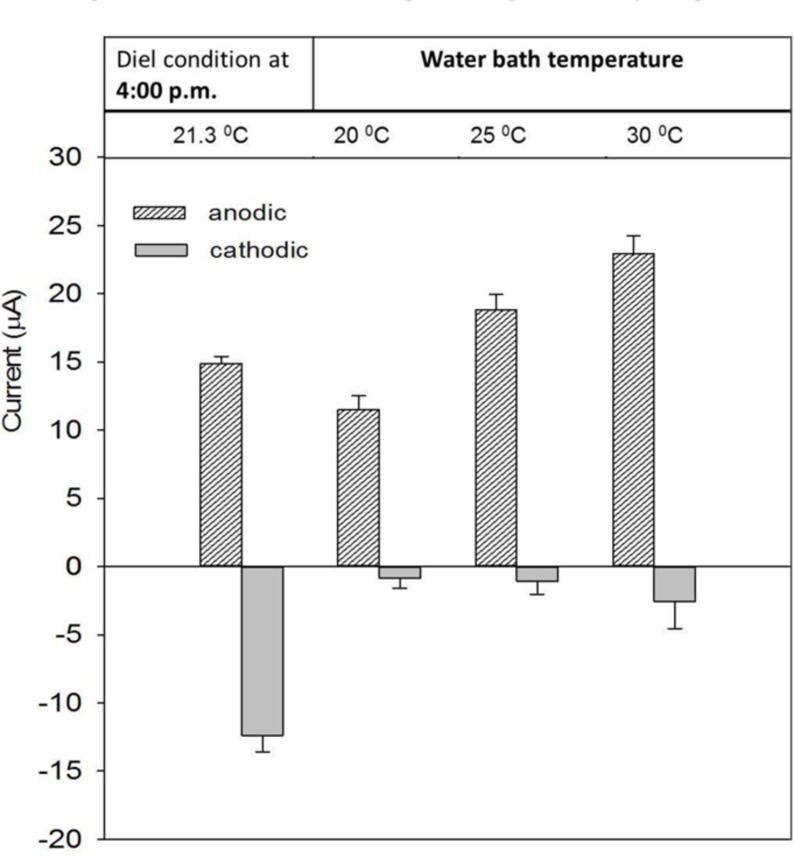
**Performance of AN and CAT currents at various temperatures**. The currents were compared with the maximum current obtained at 4:00 p.m. under the diel condition.

**Figure 4** shows that in the dark the AN current increased as temperature increased. This agrees with the observations made when the system was operated under diel temperature changes (Supplementary Figure S1). The current was highest at 4:00 p.m., when the temperature reached a maximum value of about 23°C. The effect of temperature on AN current may be due to an increase of chemical and biological activities that contribute electrons to the electrode. Because of the temperature dependence, we believe that the decrease of AN current after 15 days of operation under ambient temperature (**Figure 2A**) was caused by temperature differences owing to seasonal change.

In the dark with a constant temperature, the CAT current did not oscillate as was observed under diel cycling. Despite an increasing temperature, the current was significantly lower than the maximum obtained during diel cycling (at 4:00 p.m.; -15 μA vs. > -3.5 μA). Although the CAT current also increased with increasing temperature the change was not significant compared with the difference under a diel temperature change (Supplementary Figure S2). This suggests that the temperature change was not the main factor governing the diel cycling of CAT current.

### Effect of Light Energy on Electron Transfer Processes

**FIGURE 5:**
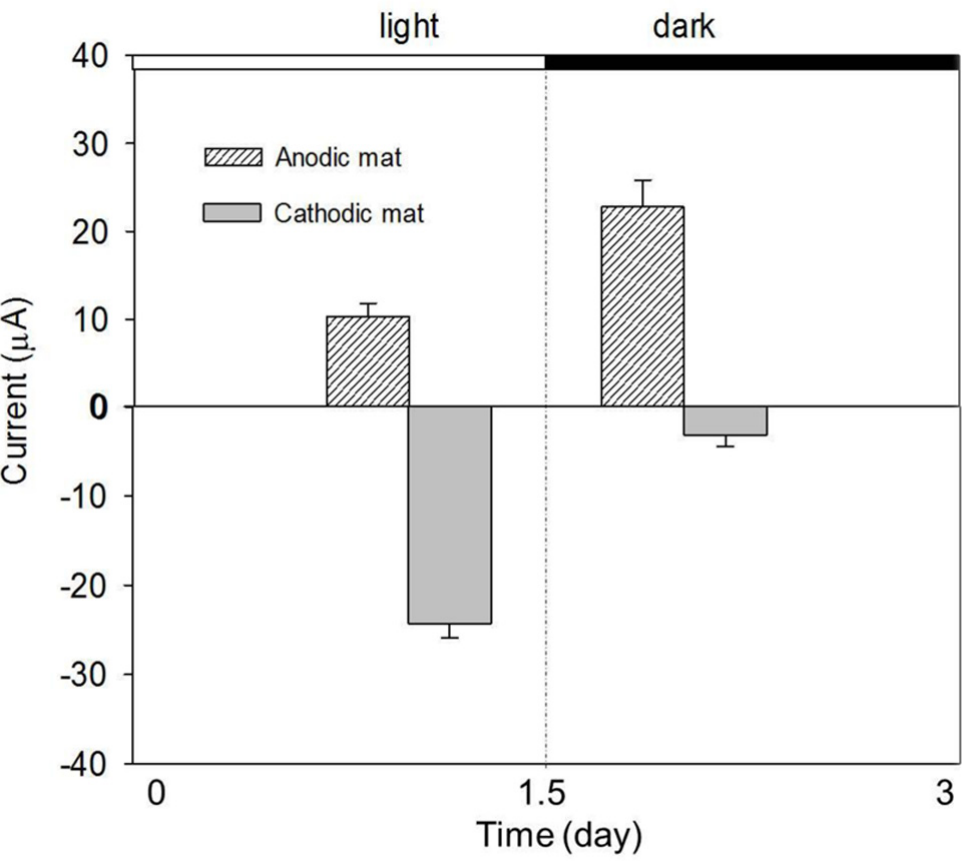
**Performance of AN and CAT currents with a change of light condition.** The AN and CAT mats showed opposite responses to the change of light condition.

The effect of the light on the AN and CAT currents was further investigated under constant temperature. **Figure 5** shows the currents obtained from the AN and CAT mats under light and dark conditions. Both the AN and the CAT current changed in response to light. The AN current increased when the system was operated in the dark (10–23 μA). In contrast, the CAT current was higher in the light than in the dark (-23 μA vs. -2.5 μA). Thus, the two exhibit an opposite responses to light.

Because of the association of light intensity and temperature, our recorded diel temperature showed that the highest temperature was reached when the light intensity was highest (at 4:00 p.m.). Thus, it is clear from the diel cycle that the highest AN current occurred when the highest light intensity was presented to the mat (**Figure 3**). This is the opposite of the observation in **Figure 5**, in which the AN current was higher in the dark than in the light. The increase of AN current during the dark might be because: when illuminated, phototrophic microbes will convert light energy to chemical energy typically stored in organic polymers such as glycogen and in the dark depolymerized to form monomers that fermented to easily respired compounds such as H_2_ and acetate. These compounds will diffuse through the mat (Des Marais, 2003; Burow et al., 2012). Fermentation products could increase sulfide production from sulfate reduction which, in consequence, increase the current caused by sulfide oxidation on electrode. Fermentation products could also be used as substrates for electrogenic bacteria which use the electrode as an electron acceptor. Thus, the inconsistency between the behavior of AN current in light/dark tests and that observed during the diel cycle suggests that a diffusion process may control the transport of fermentation products from the phototrophic layer to the bottom of the AN mat. Therefore, the highest AN current was delayed from the darkest time in the diel cycle.

The significant increase of CAT current in the light reveals that electron consumption in a CAT mat is light-dependent (**Figure 5**). This is consistent with the observation from diel cycle results in which the CAT current was highest when the highest light intensity was present. The light-dependent behavior of CAT current also explains why the CAT current in the dark was lower than that obtained at 4:00 p.m. in the diel condition in spite of increased temperature at this time (**Figure 4**). One hypothesis to explain this is that the CAT current was caused by the abiotic reduction of oxygen by electrode which diffused from the aerobic phototrophic layer. If this is the case, oxygen must have diffused through the mat fast enough that the CAT current cycle synchronized with the light intensity cycle.

### Effect of Sulfide and Acetate Additions on the Electron Transfer Process

As previously mentioned, AN current is regulated by the availability of an electron source in the surrounding environment. In this experiment, we examined the response of current to two different electron sources: sulfide and acetate. Acetate is a common end product of fermentation while sulfide is a common product of anaerobic respiration in phototrophic mats (Grotzschel et al., 2002).

**FIGURE 6:**
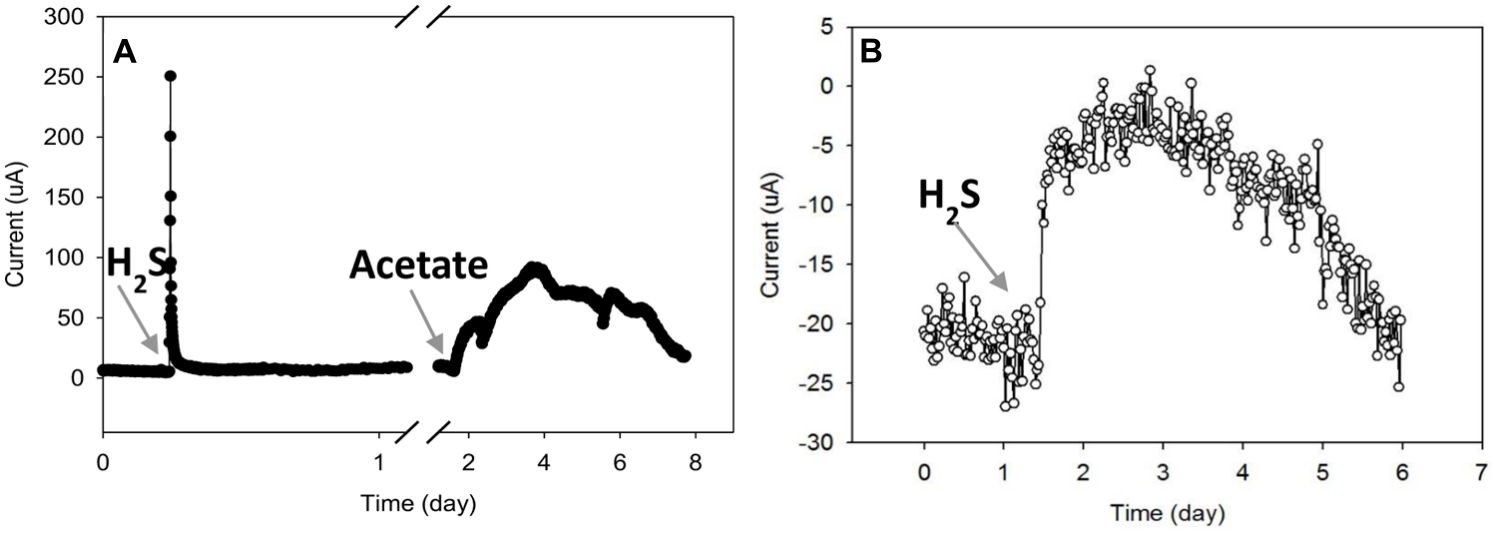
**Effects of sulfide and acetate addition to the current from **(A)** the AN mat and **(B)** the CAT mat**.

**Figure 6A** shows that the AN current increased immediately after an injection of sodium sulfide (5 mg). However, the current decreased quickly to the background value, indicating that sulfide was rapidly oxidized in this system. Since sulfide is normally produced from sulfate reduction in a microbial mat, these results demonstrate that sulfide could be abiotically oxidized by the AN electrode.

The AN current slowly increased after acetate was added (**Figure 6A**). After about 1.5 days, the current reached a maximum value of about 30 μA, then slowly decreased during the next 4 days. The gradual increasing and decreasing of AN current after acetate addition indicates that biological processes were likely involved in current generation from acetate. Acetate addition probably increased the rate of sulfate reduction at the bottom of the mat; the produced sulfide could then be oxidized on the electrode surface, driving increases in current. In addition, acetate could be used as a substrate for electrode-respiring microbes which can transfer electrons to an electrode. Thus the increase in AN current due to acetate addition may occur by both biotic and abiotic mechanisms.

The addition of the same amount of acetate (0.2 mmol) to the CAT mat did not alter current (data not shown). However, the CAT current decreased when sulfide was added (**Figure 6B**). If the CAT current was due to direct oxygen reduction on the electrode, it is possible that the introduction of sulfide reduced the oxygen available at the electrode, decreasing the current. Sulfide at high concentration is known to inhibitory or toxic to microbial metabolism including anaerobic and aerobic bacteria (Karhadkar et al., 1987; Chen et al., 2008). If these microbes are involved in the CAT current generation, it is possible that sulfide inhibited the consumption of electrons.

### Oxygen and Sulfide Profiles in the Mats over the Diel Cycle

**FIGURE 7:**
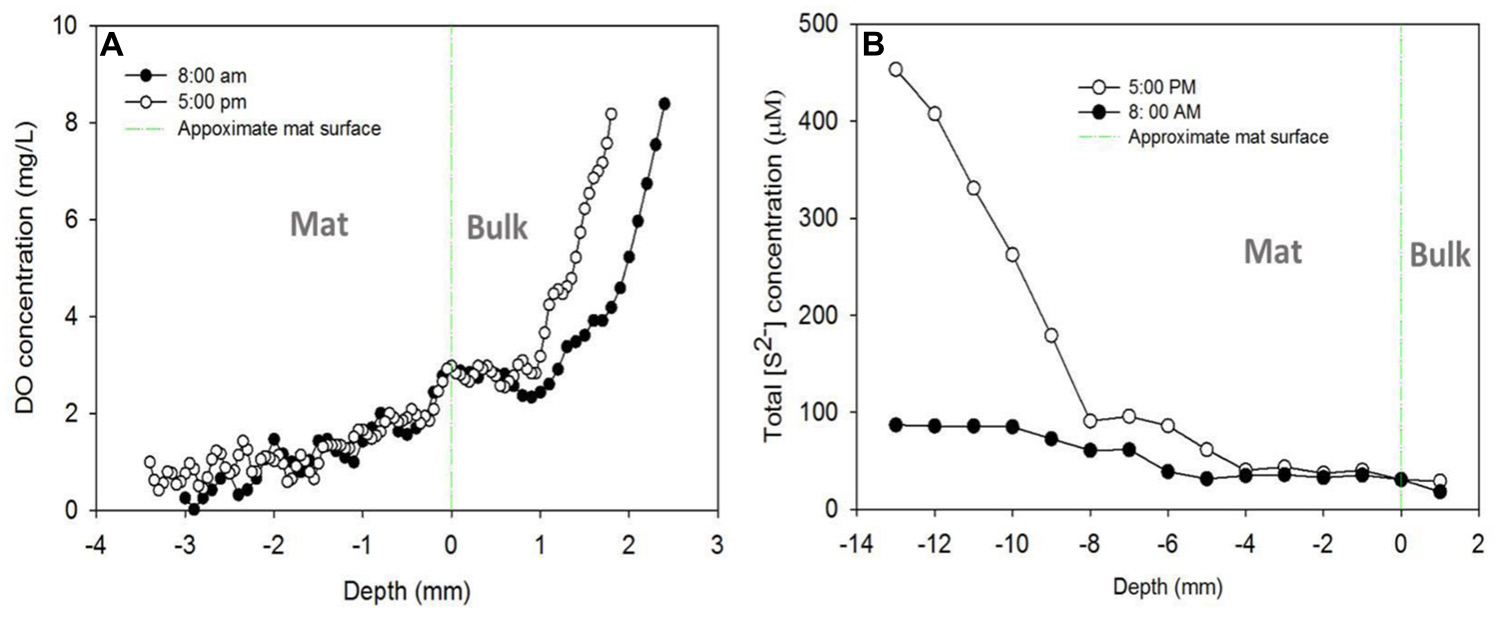
**(A)** Dissolved oxygen (DO) and **(B)** sulfide depth profiles measured from the tops of the mats at two time points (at 8:00 a.m. and 5:00 p.m.).

**Figures 7A,B** shows the DO and sulfide depth profiles measured from the tops of the mats at two time points (at 8:00 a.m. and 5:00 p.m.) during a diel cycle. We tried to measure the profiles at time points close to when the current peaks occurred, as shown in **Figure 3**. Similar DO profiles were obtained from AN mat (**Figure 7A**) and CAT mat (data not shown). The DO profile suggests that the oxygen concentration inside the mat decreased with the depth. At 3.5 mm bellow the mat surface, the DO concentration was close to zero, both for the 8:00 a.m. and 5:00 p.m. measurements. Since the thickness of the mat used in this study was 1.5–2 cm, this suggests that the environment surrounding the electrodes, which were placed at the bottom of the mat, was anoxic. To further confirm that the CAT electrode was in an anoxic environment, we switched the electrode potential of the cathode from -700 mV vs. Ag/AgCl to +300 mV vs. Ag/AgCl (i.e., to an AN condition). This switch occurred at about 4:00 p.m, when CAT current was highest (Supplementary Figure S2). Positive current (AN current) was immediately generated; suggesting that an anoxic condition at the bottom of the mat, therefore allowed electrons transfer to the electrode.

**Figure 7B** shows the change in total sulfide concentration in the mat with depth for different measurement times. The sulfide concentration increased with the depth for both 8:00 a.m. and 5:00 p.m. measurements. However, at 8:00 a.m. the increase was only 3.6-fold, from 25 μM at the surface of the mat to about 90 μM at a depth of 1.3 cm. In contrast to the profile observed at 8:00 a.m., sulfide concentration increased significantly with the depth at 5:00 p.m. The total sulfide concentration at 1.3 cm beneath the mat surface was about 18 times higher than that observed at the surface, and about 5 times higher than that obtained at the same depth at 8:00 a.m. (460 μM vs 90 μM). This sulfide profile is similar to a previous observation in a different mat (van der Meer et al., 2005; Pages et al., 2014)

Since the sulfide concentration continued to increase with depth, we left the sulfide microelectrode near the colonized electrode (at 13 mm below the mat surface) to measure sulfide concentration over the diel cycle. The diel sulfide profile shown in Supplementary Figure S3 aligns well with the oscillation of AN and CAT currents discussed above (**Figure 3**), in which the currents reached a maximum level around 5:00 p.m. and a minimum at about 8:00 a.m. This suggests that the electron transfer processes between the electrode and the mat have a strong relationship with the sulfur cycling in the mat.

### Microbial Mat Morphology and Structural Change under Different Polarized Conditions

**FIGURE 8:**
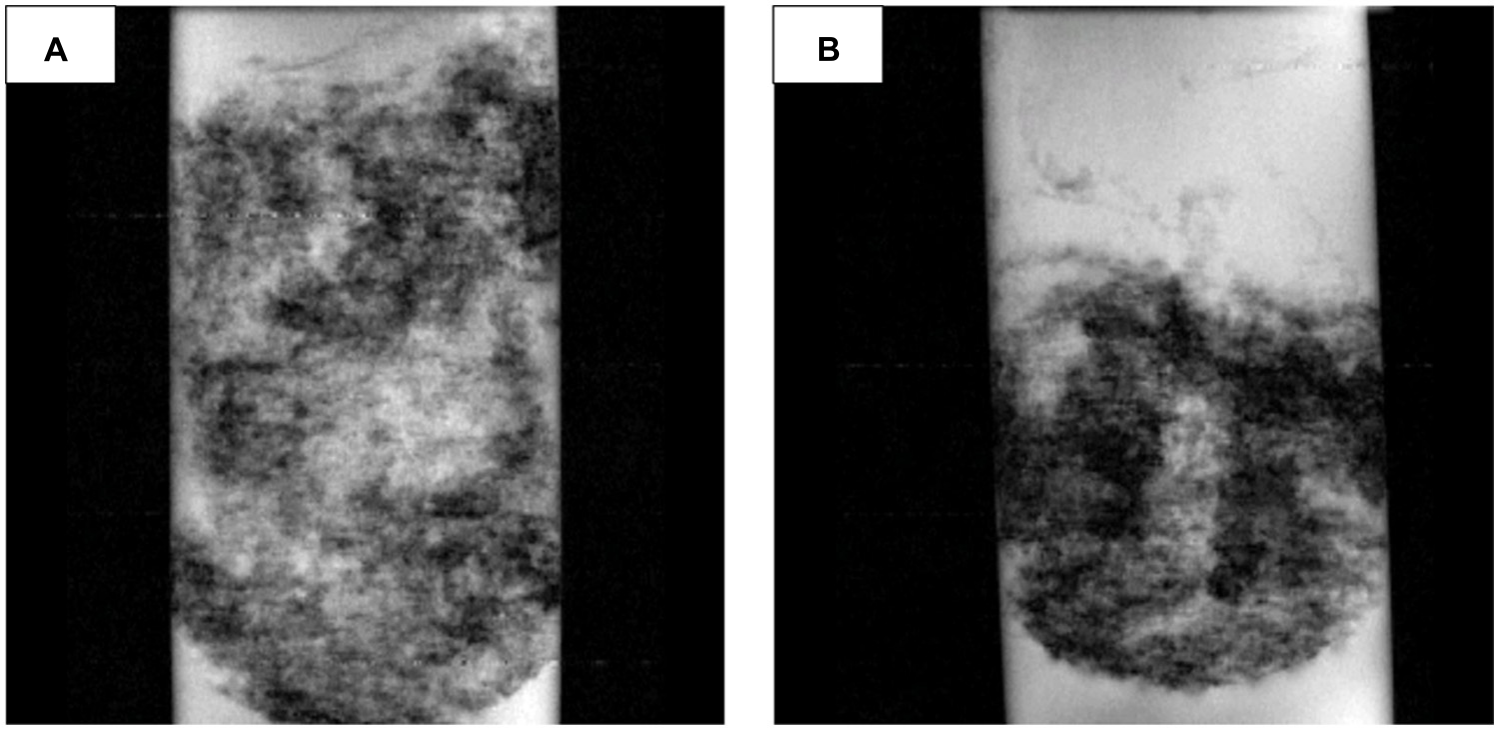
**Representative nuclear magnetic resonance images (97.7 μm × 78.1 μm resolution, 5-mm slice) of **(A)** the CAT mat and **(B)** the AN mat**.

Nuclear magnetic resonance imaging analysis showed that the internal architecture of the CAT mat system was very different from the AN mat (**Figure 8**). The darker regions in **Figure 8** qualitatively represent denser biomass and regions with less porosity. The CAT mat had dispersed biomass, and therefore was thicker and more porous than the more compact AN mat. These results corroborate the visible and tactile form of the mat samples during the harvesting period.

**FIGURE 9:**
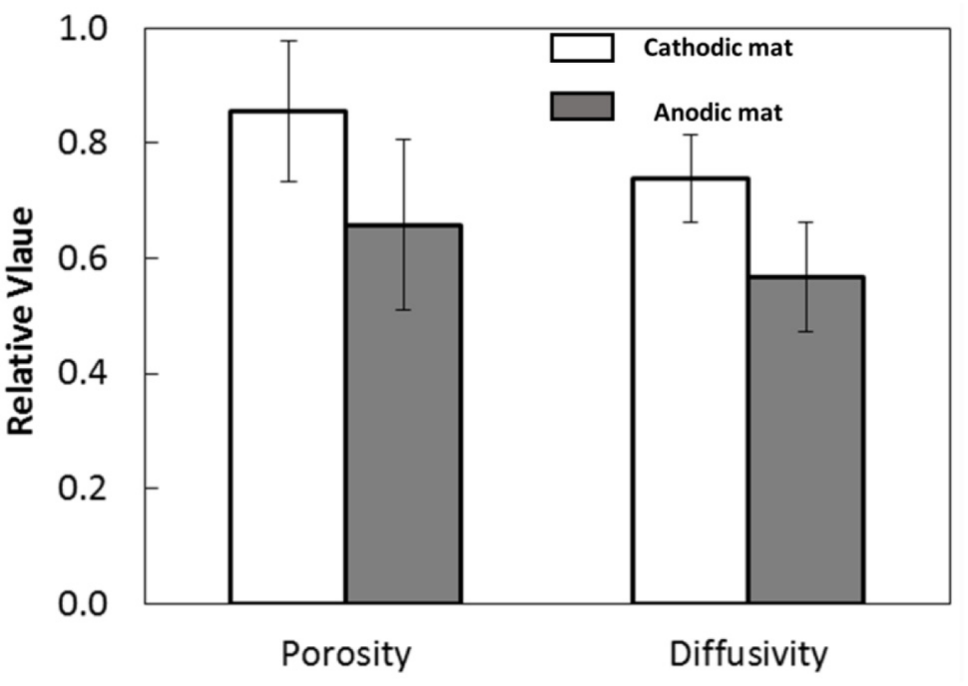
**Porosity and diffusivity of the AN and CAT mats**.

Nuclear magnetic resonance imaging showed that the quantitative porosity and diffusion coefficients were higher in the CAT mat than in the AN mat (*P*-value < 0.0001; **Figure 9**). These structural differences may have resulted from changes in microbial community composition selected under the different electrochemical conditions. Thus, this result indicates that the electrode potential regulated the development of the mat, leading to differences in morphology and structure.

The higher porosity and lower density of biomass of the CAT mat promote relatively rapid oxygen diffusion through the CAT mat, resulting in higher current. However, **Figure 2B** shows that the CAT mat was able to generate current from the initiation of the experiment, when the mat had a structure similar to that of the original sample as well as to that of the mat used in the AN system. This suggests that the electron-consuming processes in the CAT system were active in the native mat and not solely caused by the increasing porosity and diffusion coefficient of the mat.

We further used NMR spectroscopy for quantitative metabolite analysis of mat samples from our AN and CAT mats. The results were compared with data obtained from the control mat, which developed under OCP condition (OCP mat). The concentrations of major metabolites detected in the microbial mats under AN and CAT conditions are shown in Supplementary Table S1. Generally, most of the significant metabolites (with concentration >10 ug/g sample) were found at higher concentrations in the CAT mat than in the AN mat (**Table 1**). The metabolite analysis results also showed that the AN mat and OCP mat were relatively similar while the CAT mat were clearly distinguished from the other mat samples (Supplementary Table S1).

**Table 1:** Percent increase and average approximate concentration increase of primary metabolites with significant and consistently higher concentrations in the cathodic (CAT) mat than in the anodic (AN) mat.

	**June 6th collected mat**	**September 26th collected mat**	**Ave. Approx. Increase in CAT mat**
Trehalose	52.7%	162.3%	+439 μg/g
Betaine	58.8%	80.5%	+26 μg/g
Glutamate	35.1%	64.7%	+17 μg/g
Oxypurinol	143.4%	113.4%	+18 μg/g

Most of the significant metabolites—trehalose, betaine, and glutamate—are known osmolytes. Trehalose is known to be a stress molecule, protecting cells from injuries imposed by high osmolarity, heat, oxidation, and salinity (Purvis et al., 2005; Reina-Bueno et al., 2012). Similar functions have been found for betaine (McNeil et al., 1999; Kapfhammer et al., 2005) and glutamate (Feehily and Karatzas, 2013). Glutamate is also involved in the bacterial response to acid stress. Thus, the significantly higher concentrations of these compounds in the CAT mat may indicate that the CAT potential (-700 mV vs. Ag/AgCl) caused stress to the mat which led to a high response of the microbial mat community. It is possible that the addition of electrons from electrode to the mat alleviated the energy limitations allowing the mat to maintain large carbon pools of osmolytes. It is possible that some microorganisms in the mat such as acetogens and anaerobic phototrophs used electrons from the electrode to drive carbon fixations which also increased the total carbon available to the mat.

### Community Structure Changes due to Electrochemical Potential

**FIGURE 10:**
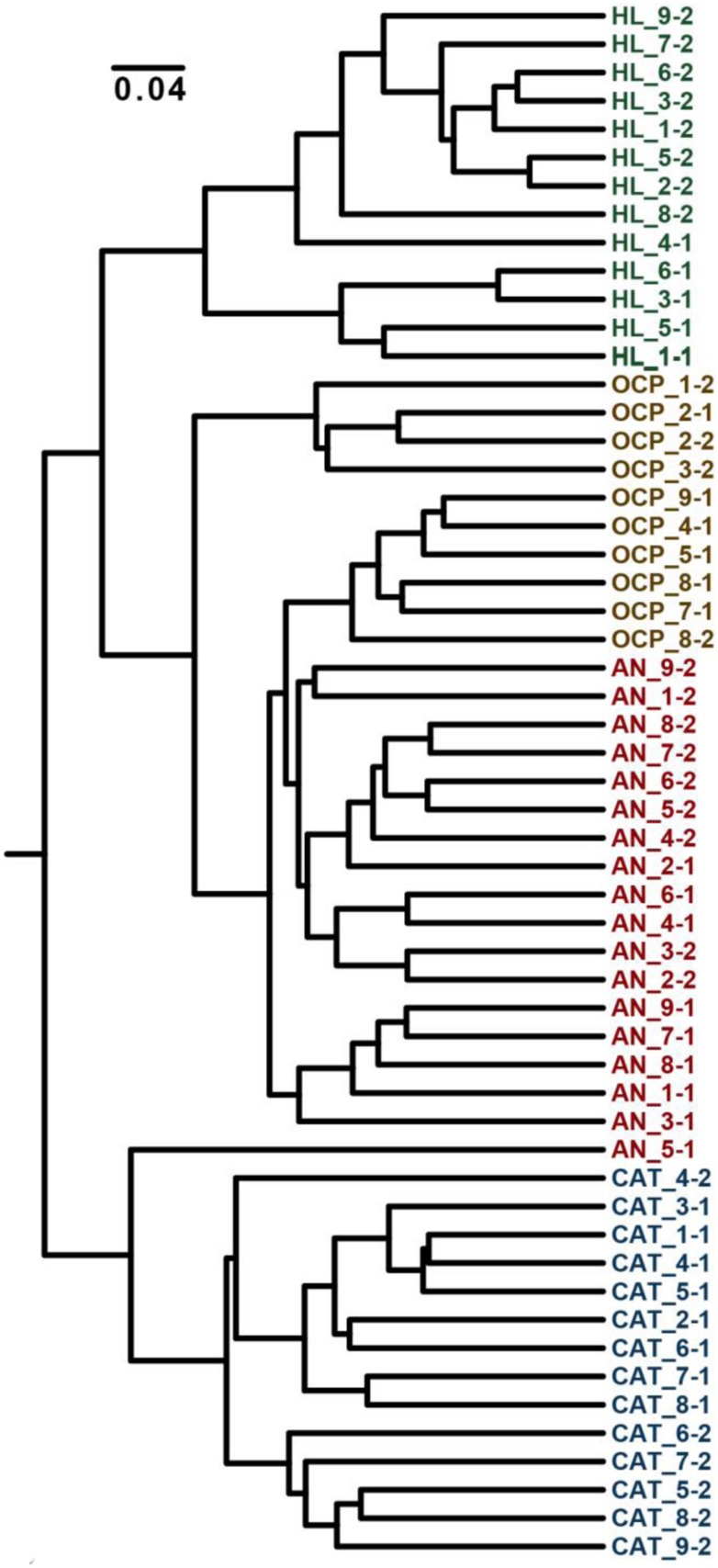
**Dendrogram of Bray–Curtis dissimilarity between mats cultivated under divergent electrochemical conditions**. Sample names denote the sample type (HL, initial mat from Hot Lake; OCP, control mat without polarization (called open circuit potential mat); AN, anodic mat; and CAT, cathodic mat), the subsample (1–9) and the sample (of two) from that condition. Mat frozen after initial collection from Hot Lake (green) forms a distinct subclade within a larger clade that encompasses open current potential (gold) and AN (red) mats. CAT mat (blue) forms a distinct clade, exhibiting a distinct community structure compared with the other conditions.

Analysis of the community structure of mats revealed that variation in the electrochemical conditions under which mats were generated significantly impacted the relative abundances of mat community members (**Figure 10**). Average within-treatment Bray–Curtis dissimilarity was 0.367 (with a pooled *SD* of 0.106) compared with 0.501 (*SD* = 0.130) for all pairwise combinations. All incubated samples were significantly different from the original mat, indicating a strong effect of electrode-based cultivation on community composition. This result was expected, considering the significant differences in environmental conditions between the field and laboratory cultivation conditions. Mat incubated under OCP formed a broad clade with AN mat; suggesting the additional effect of the anode exerted only modest impacts on microbial community structure beyond the general effects of cultivation. These data agree with the relatively small differences in mat metabolites observed between mat cultivated without polarization (OCP condition) and AN condition (Supplementary Table S1). Furthermore, this subclade was more similar to the original mat than were the CAT mats. In contrast, apart from one outlying sample of the AN mats, CAT mats formed a clade distinct from the initial, OCP, and AN mats. These community structure data are consistent with our metabolite analysis results that suggested greater changes in CAT mat metabolism compared with OCP and AN mat (Supplementary Table S1). Although it is not possible to determine whether changes in metabolism or community structure lead the CAT mat phenotype, our data strongly suggest that donation of electrons via a solid electrode is capable of strongly altering phototrophic mat morphology, community composition, and metabolic function.

## Conclusion

We quantified the diel variations of electron transfer rates to and from the solid electrode in the mat electrochemical systems. We observed the diel cycling of current in which the lowest and highest magnitudes of AN current occurred simultaneously with those of CAT current. We also characterized the influence of physicochemical conditions (temperature, light, sulfide, and DO gradients) within the phototrophic mat upon electron transfer processes. AN current was higher in the dark and it increased with temperature. In contrast, CAT current increased with the illumination but did not change significantly with increased temperature. Comparing the behavior of AN current under constant light and dark conditions vs. diel-cycling mats revealed that AN current reached maximum under photic conditions under diel cycling conditions but was greater under constant dark conditions. This suggested diffusion control of the fermentable substrates from the photic layer to the bottom of the AN mat. We propose that the generated diel cycle data can be used to develop model of energy flow in the mat to predict diffusion controls. DO and sulfide gradients over diel cycle suggested that the current cycles of CAT and AN mats related to sulfur cycling in the mat. The congruence of community structure data with metabolite profiles suggested that variation in the electrochemical conditions under which mats were generated significantly impacted the relative abundances of mat members and mat metabolism. It remains unclear whether the electron transfer to and from the electrode to the mat was mediated by microbial metabolism of selectively enriched community on the electrode or was caused by abiotic reactions on electrode. These findings suggest that it is possible to electrochemically regulate the morphology, community composition, and activities of phototrophic mats and may represent a new paradigm for designing and manipulating microbial communities (Fredrickson, 2015).

## Conflict of Interest Statement

The authors declare that the research was conducted in the absence of any commercial or financial relationships that could be construed as a potential conflict of interest.

## References

[B1] Babauta J. T. Atci E. Ha P. T. Lindemann S. R. Ewing T. Call D. R. (2014). Localized electron transfer rates and microelectrode-based enrichment of microbial communities within a phototrophic microbial mat. *Front. Microbiol.* 5:11 10.3389/fmicb.2014.00011 PMC3902354 24478768

[B2] Babauta J. T. Nguyen H. D. Istanbullu O. Beyenal H. (2013). Microscale gradients of oxygen, hydrogen peroxide, and ph in freshwater cathodic biofilms. *Chemsuschem* 6 1252–1261. 10.1002/cssc.201300019 23766295 PMC4247834

[B3] Badalamenti J. P. Torres C. I. Krajmalnik-Brown R. (2013). Light-responsive current generation by phototrophically enriched anode biofilms dominated by green sulfur bacteria. *Biotechnol. Bioeng.* 110 1020–1027. 10.1002/bit.24779 23124549

[B4] Bender J. Phillips P. (2004). Microbial mats for multiple applications in aquaculture and bioremediation. *Bioresour. Technol.* 94 229–238. 10.1016/j.biortech.2003.12.016 15182828

[B5] Bose A. Gardel E. J. Vidoudez C. Parra E. A. Girguis P. R. (2014). Electron uptake by iron-oxidizing phototrophic bacteria. *Nat. Commun.* 5:3391 10.1038/ncomms4391 24569675

[B6] Bray J. R. Curtis J. T. (1957). An ordination of the upland forest communities of southern wisconsin. *Ecol. Monogr.* 27 326–349. 10.2307/1942268

[B7] Burow L. C. Woebken D. Bebout B. M. McMurdie P. J. Singer S. W. Pett-Ridge J. (2012). Hydrogen production in photosynthetic microbial mats in the Elkhorn Slough estuary. *Monterey Bay. ISME J.* 6 863–874. 10.1038/ismej.2011.142 22011721 PMC3309353

[B8] Caporaso J. G. Lauber C. L. Walters W. A. Berg-Lyons D. Huntley J. Fierer N. (2012). Ultra-high-throughput microbial community analysis on the Illumina HiSeq and MiSeq platforms. *ISME J.* 6 1621–1624. 10.1038/ismej.2012.8 22402401 PMC3400413

[B9] Caporaso J. G. Lauber C. L. Walters W. A. Berg-Lyons D. Lozupone C. A. Turnbaugh P. J. (2011). Global patterns of 16S rRNA diversity at a depth of millions of sequences per sample. *Proc. Natl. Acad. Sci. U.S.A.* 108 4516–4522. 10.1073/pnas.1000080107 20534432 PMC3063599

[B10] Chen Y. Cheng J. J. Creamer K. S. (2008). Inhibition of anaerobic digestion process: a review. *Bioresour. Technol.* 99 4044–4064. 10.1016/j.biortech.2007.01.057 17399981

[B11] Clauwaert P. Van der Ha D. Boon N. Verbeken K. Verhaege M. Rabaey K. (2007). Open air biocathode enables effective electricity generation with microbial fuel cells. *Environ. Sci. Technol.* 41 7564–7569. 10.1021/es0709831 18044542

[B12] Debuy S. Pecastaings S. Bergel A. Erable B. (2015). Oxygen-reducing biocathodes designed with pure cultures of microbial strains isolated from seawater biofilms. *Int. Biodeterior. Biodegradation* 103 16–22. 10.1016/j.ibiod.2015.03.028

[B13] Decker K. L. M. Potterb C. S. Beboutc B. M. Des Maraisc D. J. Carpenterd S. Discipulo M. (2005). Mathematical simulation of the diel O, S, and C biogeochemistry of a hypersaline microbial mat. *Fems Microbiol. Ecol.* 52 377–395. 10.1016/j.femsec.2004.12.005 16329922

[B14] Des Marais D. J. (1995). The biogeochemistry of hypersaline microbial mats. *Adv. Microb. Ecol.* 14 251–274. 10.1007/978-1-4684-7724-5_6 11539110

[B15] Des Marais D. J. (2003). Biogeochemistry of hypersaline microbial mats illustrates the dynamics of modern microbial ecosystems and the early evolution of the biosphere. *Biol. Bull-Us* 204 160–167. 10.2307/1543552 12700147

[B16] Feehily C. Karatzas K. A. G. (2013). Role of glutamate metabolism in bacterial responses towards acid and other stresses. *J. Appl. Microbiol.* 114 11–24. 10.1111/j.1365-2672.2012.05434.x 22924898

[B17] Finke N. Jorgensen B. B. (2008). Response of fermentation and sulfate reduction to experimental temperature changes in temperate and Arctic marine sediments. *ISME J.* 2 815–829. 10.1038/ISMEJ.2008.20 18309360

[B18] Fredrickson J. K. (2015). ECOLOGY. Ecological communities by design. *Science* 348 1425–1427. 10.1126/science.aab0946 26113703

[B19] Frund C. Cohen Y. (1992). Diurnal cycles of sulfate reduction under oxic conditions in cyanobacterial mats. *Appl. Environ. Microb.* 58 70–77. 10.1128/aem.58.1.70-77.1992 PMC195174 16348641

[B20] Gregory K. B. Bond D. R. Lovley D. R. (2004). Graphite electrodes as electron donors for anaerobic respiration. *Environ. Microbiol.* 6 596–604. 10.1111/j.1462-2920.2004.00593.x 15142248

[B21] Grotzschel S. Abed R. M. M. de Beer D. (2002). Metabolic shifts in hypersaline microbial mats upon addition of organic substrates. *Environ. Microbiol.* 4 683–695. 10.1046/j.1462-2920.2002.00356.x 12460276

[B22] Guerrero R. Piqueras M. Berlanga M. (2002). Microbial mats and the search for minimal ecosystems. *Int. Microbiol.* 5 177–188. 10.1007/s10123-002-0094-8 12497183

[B23] Jeroschewski P. Steuckart C. Kuhl M. (1996). An amperometric microsensor for the determination of H2S in aquatic environments. *Anal. Chem.* 68 4351–4357. 10.1021/ac960091b

[B24] Kapfhammer D. Karatan E. Pflughoeft K. J. Watnick P. I. (2005). Role for glycine betaine transport in *Vibrio cholerae* osmoadaptation and biofilm formation within microbial communities. *Appl. Environ. Microbiol.* 71 3840–3847. 10.1128/AEM.71.7.3840-3847.2005 16000796 PMC1169069

[B25] Karhadkar P. P. Audic J. M. Faup G. M. Khanna P. (1987). Sulfide and sulfate inhibition of methanogenesis. *Water Res.* 21 1061–1066. 10.1016/0043-1354(87)90027-3

[B26] Lewandowski Z. Beyenal H. (2014). *Fundamentals of Biofilm Research.* Abingdon: Taylor & Francis.

[B27] Lindemann S. R. Moran J. J. Stegen J. C. Renslow R. S. Hutchison J. R. Cole J. K. (2013). The epsomitic phototrophic microbial mat of Hot Lake, Washington: community structural responses to seasonal cycling. *Front. Microbiol.* 4:323 10.3389/fmicb.2013.00323 PMC3826063 24312082

[B28] Logan B. E. (2009). Exoelectrogenic bacteria that power microbial fuel cells. *Nat. Rev. Microbiol.* 7 375–381. 10.1038/nrmicro2113 19330018

[B29] Lovley D. R. (2008). The microbe electric: conversion of organic matter to electricity. *Curr. Opin. Biotech* 19 564–571. 10.1016/j.copbio.2008.10.005 19000760

[B30] Lovley D. R. Nevin K. P. (2011). A shift in the current: new applications and concepts for microbe-electrode electron exchange. *Curr. Opin. Biotech.* 22 441–448. 10.1016/j.copbio.2011.01.009 21333524

[B31] Marshall C. W. Ross D. E. Fichot E. B. Norman R. S. May H. D. (2012). Electrosynthesis of commodity chemicals by an autotrophic microbial community. *Appl. Environ. Microb.* 78 8412–8420. 10.1128/AEM.02401-12 PMC3497389 23001672

[B32] McNeil S. D. Nuccio M. L. Hanson A. D. (1999). Betaines and related osmoprotectants. Targets for metabolic engineering of stress resistance. *Plant Physiol.* 120 945–949. 10.1104/pp.120.4.945 10444077 PMC1539222

[B33] Moran J. J. Doll C. G. Bernstein H. C. Renslow R. S. Cory A. B. Hutchison J. R. (2014). Spatially tracking C-13-labelled substrate (bicarbonate) accumulation in microbial communities using laser ablation isotope ratio mass spectrometry. *Environ. Microbiol. Rep.* 6 786–791. 10.1111/1758-2229.12211 25155264

[B34] Nevin K. P. Hensley S. A. Franks A. E. Summers Z. M. Ou J. Woodard T. L. (2011). Electrosynthesis of organic compounds from carbon dioxide is catalyzed by a diversity of acetogenic microorganisms. *Appl. Environ. Microb.* 77 2882–2886. 10.1128/AEM.02642-10 PMC3126412 21378039

[B35] Paerl H. W. Pinckney J. L. Steppe T. F. (2000). Cyanobacterial–bacterial mat consortia: examining the functional unit of microbial survival and growth in extreme environments. *Environ. Microbiol.* 2 11–26. 10.1046/j.1462-2920.2000.00071.x 11243256

[B36] Pages A. Welshb D. T. Teasdaleb P. R. Gricea K. Vacherc M. Bennettb W. W. (2014). Diel fluctuations in solute distributions and biogeochemical cycling in a hypersaline microbial mat from Shark Bay. WA. *Mar. Chem.* 167 102–112. 10.1016/j.marchem.2014.05.003

[B37] Purvis J. E. Yomano L. P. Ingram L. O. (2005). Enhanced trehalose production improves growth of *Escherichia coli* under osmotic stress. *Appl. Environ. Microb.* 71 3761–3769. 10.1128/AEM.71.7.3761-3769.2005 PMC1168978 16000787

[B38] Rabaey K. Rodríguez J. Blackall L. L. Keller J. Gross P. Batstone D. (2007). Microbial ecology meets electrochemistry: electricity-driven and driving communities. *ISME J.* 1 9–18. 10.1038/ismej.2007.4 18043609

[B39] Rabaey K. Van de Sompel K. Maignien L. Boon N. Aelterman P. Clauwaert P. (2006). Microbial fuel cells for sulfide removal. *Environ. Sci. Technol.* 40 5218–5224. 10.1021/es060382u 16999092

[B40] Reina-Bueno M. Argandoña M. Salvador M. Rodríguez-Moya J. Iglesias-Guerra F. Csonka L. N. (2012). Role of trehalose in salinity and temperature tolerance in the model halophilic bacterium chromohalobacter salexigens. *PLoS ONE* 7:e33587 10.1371/journal.pone.0033587 PMC3308980 22448254

[B41] Renslow R. Babauta J. Kuprat A. Schenk J. Ivory C. Fredrickson J. (2013). Modeling biofilms with dual extracellular electron transfer mechanisms. *Phys. Chem. Chem. Phys.* 15 19262–19283. 10.1039/c3cp53759e 24113651 PMC3868370

[B42] Renslow R. Donovan C. Shim M. Babauta J. Nannapaneni S. Schenk J. (2011). Oxygen reduction kinetics on graphite cathodes in sediment microbial fuel cells. *Phys. Chem. Chem. Phys.* 13 21573–21584. 10.1039/c1cp23200b 22052235 PMC3551589

[B43] Rosenbaum M. Aulenta F. Villano M. Angenent L. T. (2011). Cathodes as electron donors for microbial metabolism: which extracellular electron transfer mechanisms are involved? *Bioresour. Technol.* 102 324–333. 10.1016/j.biortech.2010.07.008 20688515

[B44] Schloss P. D. Westcott S. L. Ryabin T. Hall J. R. Hartmann M. Hollister E. B. (2009). Introducing mothur: open-Source, platform-independent, community-supported software for describing and comparing microbial communities. *Appl. Environ. Microb.* 75 7537–7541. 10.1128/AEM.01541-09 PMC2786419 19801464

[B45] Su M. Jiang Y. Li D. (2013). Production of acetate from carbon dioxide in bioelectrochemical systems based on autotrophic mixed culture. *J. Microbiol. Biotechnol.* 23 1140–1146. 10.4014/jmb.1304.04039 23727797

[B46] Tremblay P. L. Zhang T. (2015). Electrifying microbes for the production of chemicals. *Front. Microbiol.* 6:201 10.3389/fmicb.2015.00201 PMC4356085 25814988

[B47] Ueki T. Nevin K. P. Woodard T. L. Lovley D. R. (2014). Converting carbon dioxide to butyrate with an engineered strain of *Clostridium ljungdahlii*. *MBio* 5:e1636-14 10.1128/mBio.01636-14 PMC4212834 25336453

[B48] van der Meer M. T. J. Schouten S. Bateson M. M. Nübel U. Wieland A. Kühl M. (2005). Diel variations in carbon metabolism by green nonsulfur-like bacteria in alkaline siliceous hot spring microbial mats from Yellowstone National Park. *Appl. Environ. Microb.* 71 3978–3986. 10.1128/AEM.71.7.3978-3986.2005 PMC1168979 16000812

[B49] Villano M. Aulenta F. Ciucci C. Ferri T. Giuliano A. Majone M. (2010). Bioelectrochemical reduction of CO2 to CH4 via direct and indirect extracellular electron transfer by a hydrogenophilic methanogenic culture. *Bioresour. Technol.* 101 3085–3090. 10.1016/j.biortech.2009.12.077 20074943

[B50] Wagner R. C. Call D. F. Logan B. E. (2010). Optimal set anode potentials vary in bioelectrochemical systems. *Environ. Sci. Technol.* 44 6036–6041. 10.1021/es101013e 20704197

