# Supplementary material for: Regulation of electron transfer processes affects phototrophic mat structure and activity
Source: Front Microbiol. 2015 Sep 3;6:909. doi: 10.3389/fmicb.2015.00909 (PMC4558538; doi:10.3389/fmicb.2015.00909)
Supplement: Supplementary file 2 [file Supplemental_Data_1.DOCX]

Supplementary Information

**Regulation of electron transfer processes affects phototrophic mat structure and activity**

Phuc T. Ha^1^, Ryan S. Renslow^2^, Erhan Atci^1^, , Patrick N. Reardon^2^, Stephen R. Lindemann^3^, James K. Fredrickson^3^, Douglas R. Call^4^ and Haluk Beyenal^1^

^1^ The Gene and Linda Voiland School of Chemical Engineering and Bioengineering, Washington State University, Pullman, WA, USA

^2^ Environmental Molecular Sciences Laboratory, Pacific Northwest National Laboratory, Richland, WA, USA

^3^ Biological Sciences Division, Pacific Northwest National Laboratory, Richland, WA, USA

^4^ Paul G. Allen School for Global Animal Health, Washington State University, Pullman, WA, USA

**Correspondence:**

Dr. Haluk Beyenal

The Gene and Linda Voiland School of Chemical Engineering and Bioengineering

Washington State University

P.O. Box 642710

Pullman, WA, 99164-2710

[beyenal@wsu.edu](mailto:beyenal@wsu.edu)

Running title: Electron transfer in microbial mats

**Supplementary Material and Methods**

***Nuclear magnetic resonance imaging***: Pulsed-field gradient nuclear magnetic resonance (PFG-NMR) was used to determine porosity and diffusion coefficients in both anodic and cathodic microbial mat samples. The NMR experiments were conducted at 500.40 MHz for proton (^1^H) detection using an 89-mm wide bore 11.7-T magnet with a Bruker Avance III digital NMR spectrometer (Bruker Instruments, Billerica, MA). Bruker ParaVision 5.1 imaging software was used to collect and process the data. FLASH (fast low angle shot, mic_flash) magnetic resonance imaging was used for orientation and visualization of the mat samples. Each sample was placed in a 15 mm NMR tube on a support bed of 2% agar gel. Experiments performed included 2D magnetic resonance imaging, diffusion tensor imaging for determining diffusion coefficients, and chemical shift selective imaging for generating porosity measurements.

The diffusion tensor imaging method (DtiStandard) was run with a 1000 ms repetition time, an echo time of 17.2 ms, and 8 averages. The pulse gradient width (∂) was 2 ms and the diffusion time interval (∆) was 10 ms. Signal intensity was measured for eight independent *b*-factors – 0, 200, 250, 350, 500, 700, 950, and 1250 s/mm^2^. The field of view dimensions were 25 mm in the microbial mat depth direction and 20 mm in the perpendicular direction, with a 5 mm thick slice. A total of 256 complex points were sampled in the depth direction, with 64 phase encoding steps, for an in-plane resolution of 97.7 μm by 312.5 μm. The total experiment time per experiment was 60 min. Diffusion coefficients were calculated using semi-logarithmic regression of the *b*-factor-dependent intensity value of each measurement pixel in accord with the Block-Torrey differential equation. Average diffusion coefficients were generated by averaging and performing statistical analysis on the diffusion coefficients in the middle of each mat sample. PFG-NMR measures the diffusion coefficient of water. The measured diffusion coefficient was normalized by dividing the value at a local point to the diffusion coefficient in the bulk. This normalized diffusion coefficient is called the relative effective diffusion coefficient.

The chemical shift selective imaging method (mic_chess) was run with a 15000 ms repetition time to allow for complete relaxation of ^1^H nuclei, with an echo time of 6.9 ms. The excitation radio frequency pulses were centered on water with a bandwidth of 1500 Hz. The field of view and slice were identical to the diffusion tensor imaging method. A total of 250 complex points were sampled in the depth direction, with 200 phase encoding steps, for an in-plane resolution of 100 μm by 100 μm. The total experiment time per experiment was 50 min. Average mat porosity was generated by averaging and performing statistical analysis on the signal in the middle of each mat sample. The average signal was normalized by dividing by the signal of pure water located above the mat samples, yielding an average porosity measurement for each mat sample.

**Supplementary Results and Discussion**


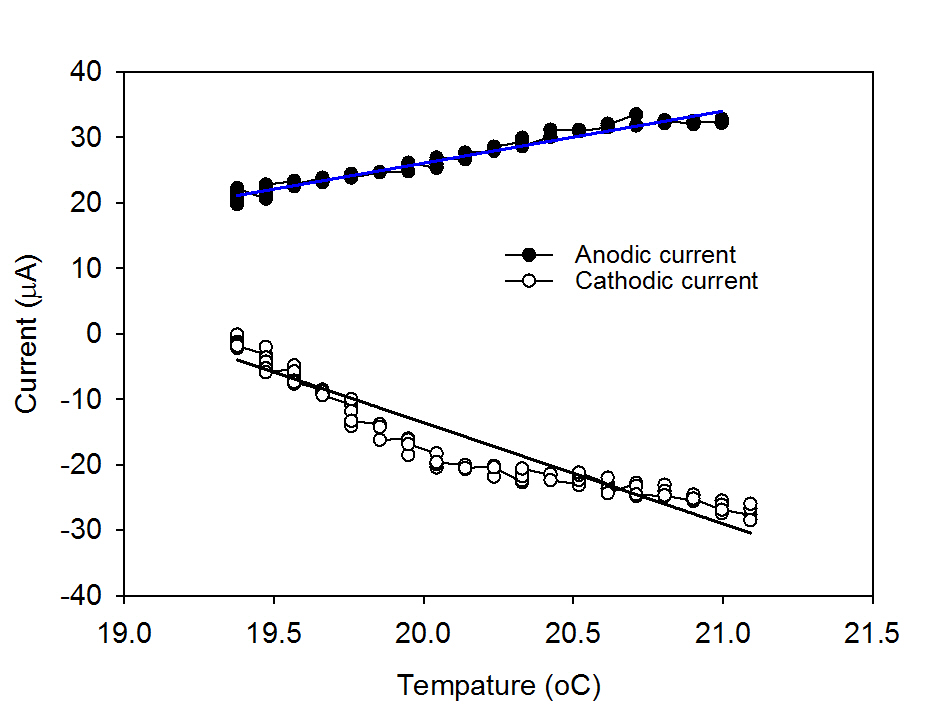


Figure S1. Relationship between current with the temperature change under diel condition. The plotted current and temperature were recorded between 8:00 am to 5:00 pm when anodic current and cathodic current changed from minimum value to maximum value. At this time, the systems were illuminated by natural sun light. Both anodic and cathodic current showed a linear relationship with temperature. The large shift of cathodic current was obtained when temperature increased from 19.4 to 21.1 ^o^C under illuminated condition (approximately -2 µA to approximately -30 µA).


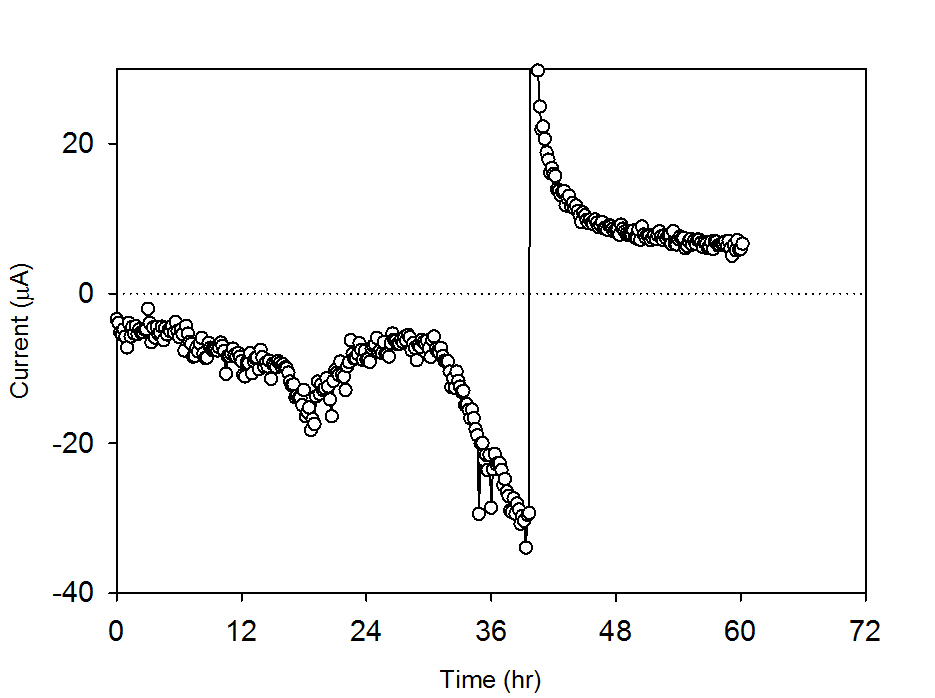


Figure S2: Current change after switching the electrode potential from -700 mV vs Ag/AgCl (cathodic) to +300 mV vs Ag/AgCl (anodic). The change was made at about 4:00 pm when the cathodic current was at maximum level. The positive current was generated immediately after changing the potential suggesting that the condition at the bottom of mat wass anoxic, allowing for electron transfer to the electrode.


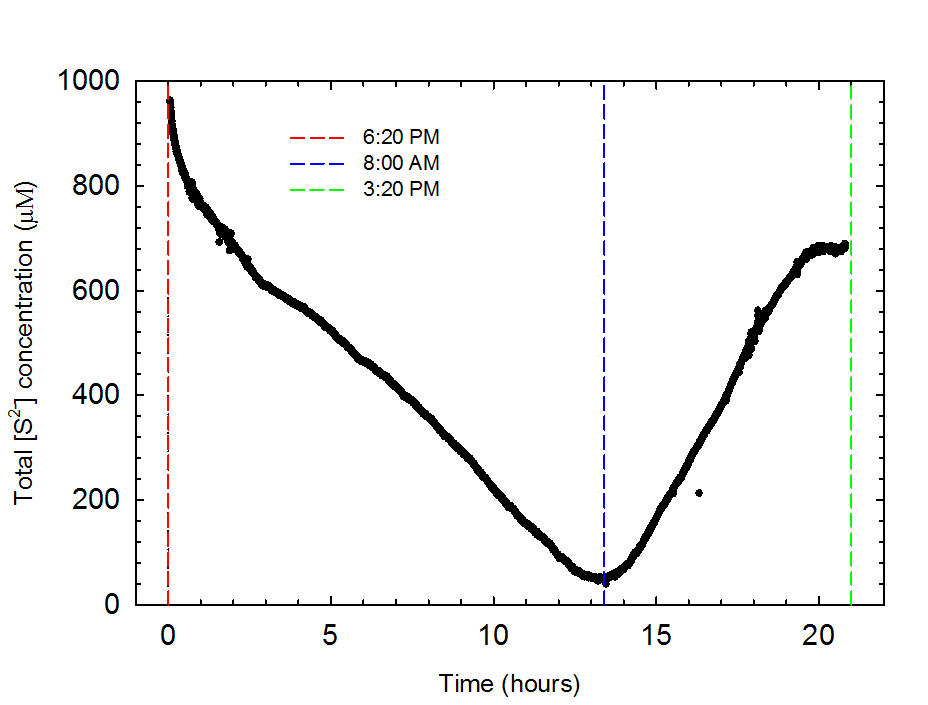


Figure S3. Sulfide concentration in the mat over time. The sulfide microelectrode was placed near the electrode (1.3 cm depth from mat surface) to measure sulfide concentration change over the diel time cycle. This sulfide profile correlates with the diel cycles of current in which the current reached minimum value at around 8AM and maximum value at about 4:00 pm

Table S1: The variation of major metabolites in different microbial mat samples (µg/g of dry weigh mat)

| **Metabolites products** | **June 6^th^ collected mat** | | | **September 26^th^ collected mat** | | |
| --- | --- | --- | --- | --- | --- | --- |
|  | **Anodic**  **mat** | **Cathodic**  **mat** | **OCP mat** | **Anodic**  **mat** | **Cathodic**  **mat** | **OCP**  **mat** |
| **Trehalose** | 440.1 | 671.8 | 450.4 | 397.9 | 1043.9 | 604.9 |
| **Acetate** | 103.1 | 109.6 | 76.8 | 78.9 | 74.2 | 58.2 |
| **Betaine** | 32.3 | 51.2 | 30.3 | 41.6 | 75.1 | 32.3 |
| **Glutamate** | 30.4 | 41.0 | 35.7 | 35.6 | 58.6 | 24.2 |
| **Sucrose** | 38.6 | 91.4 | 23.7 | 25.6 | 21.1 | 6.5 |
| **Oxypurinol** | 10.8 | 26.3 | 17.4 | 18.4 | 39.4 | 17.1 |
| **Malonate** | 8.4 | 13.5 | 8.6 | 14.0 | 18.5 | 11.4 |
| **3-Hydroxybutyrate** | 12.2 | 5.2 | 4.3 | 7.8 | 23.8 | 8.4 |
| **Trimethylamine N-oxide** | 3.4 | 4.3 | 5.1 | 5.0 | 9.4 | 5.1 |
| **Formate** | 5.5 | 5.1 | 4.1 | 3.9 | 6.0 | 2.8 |
| **Alanine** | 0.9 | 2.2 | 1.3 | 2.3 | 5.0 | 2.8 |
| **Propionate** | 2.8 | 2.7 | 1.6 | 2.3 | 1.9 | 1.3 |
| **Acetone** | 2.2 | 1.9 | 1.4 | 1.9 | 1.6 | 1.4 |
| **Methylamine** | 1.2 | 1.0 | 0.8 | 0.9 | 0.8 | 0.7 |
